# Moderately high frequency ventilation with a conventional ventilator allows reduction of tidal volume without increasing mean airway pressure

**DOI:** 10.1186/2197-425X-2-13

**Published:** 2014-05-09

**Authors:** Ricardo Luiz Cordioli, Marcelo Park, Eduardo Leite Vieira Costa, Susimeire Gomes, Laurent Brochard, Marcelo Britto Passos Amato, Luciano Cesar Pontes Azevedo

**Affiliations:** Research and Education Institute, Hospital Sírio-Libanês, Rua Dona Adma Jafet, 91, Bela Vista, São Paulo 01308-050 Brazil; Laboratório de Investigação Médica 09, Heart Institute (InCor), Hospital das Clínicas da Faculdade de Medicina da Universidade de São Paulo, Av. Rebouças, 381-Jardim Paulista, São Paulo, São Paulo 05401-000 Brazil; Emergency Medicine Department, Faculdade de Medicina da Universidade de São Paulo, Rua Dona Adma Jafet, 91, Bela Vista, São Paulo 01308-050 Brazil; Department of Adult Intensive Care, Intensive Care Unit, Hospital Israelita Albert Einstein, Av. Albert Einstein, 627, Morumbi, São Paulo 05652-900 Brazil; St Michael’s Hospital, Toronto, University of Toronto, 30 Bond Street, Toronto, Ontario M5B 1W8 Canada; Intensive Care Unit, Hospital Alemão Oswaldo Cruz, Rua João Julião, 331, Bela Vista, São Paulo 01323-903 Brazil

**Keywords:** Mechanical ventilation, Acute lung injury, Critical care unit, Protective ventilation, Acute respiratory distress syndrome, High-frequency ventilation

## Abstract

**Background:**

The aim of this study was to explore if positive-pressure ventilation delivered by a conventional ICU ventilator at a moderately high frequency (HFPPV) allows a safe reduction of tidal volume (*V*_T_) below 6 mL/kg in a porcine model of severe acute respiratory distress syndrome (ARDS) and at a lower mean airway pressure than high-frequency oscillatory ventilation (HFOV).

**Methods:**

This is a prospective study. In eight pigs (median weight 34 [29,36] kg), ARDS was induced by pulmonary lavage and injurious ventilation. The animals were ventilated with a randomized sequence of respiratory rates: 30, 60, 90, 120, 150, followed by HFOV at 5 Hz. At each step, *V*_T_ was adjusted to allow partial pressure of arterial carbon dioxide (PaCO_2_) to stabilize between 57 and 63 mmHg. Data are shown as median [P25th,P75th].

**Results:**

After lung injury, the PaO_2_/FiO_2_ (P/F) ratio was 92 [63,118] mmHg, pulmonary shunt 26 [17,31]%, and static compliance 11 [8,14] mL/cmH_2_O. Positive end-expiratory pressure (PEEP) was 14 [10,17] cmH_2_O. At 30 breaths/min, *V*_T_ was higher than 6 (7.5 [6.8,10.2]) mL/kg, but at all higher frequencies, *V*_T_ could be reduced and PaCO_2_ maintained, leading to reductions in plateau pressures and driving pressures. For frequencies of 60 to 150/min, *V*_T_ progressively fell from 5.2 [5.1,5.9] to 3.8 [3.7,4.2] mL/kg (*p* < 0.001). There were no detrimental effects in terms of lung mechanics, auto-PEEP generation, hemodynamics, or gas exchange. Mean airway pressure was maintained constant and was increased only during HFOV.

**Conclusions:**

During protective mechanical ventilation, HFPPV delivered by a conventional ventilator in a severe ARDS swine model safely allows further tidal volume reductions. This strategy also allowed decreasing airway pressures while maintaining stable PaCO_2_ levels.

## Background

Acute respiratory distress syndrome (ARDS) is a common cause of mortality and morbidity in critically ill patients [1]. Although indispensable in the support of ARDS patients, artificial ventilation involves the application of mechanical forces to the lung parenchyma that can further induce injury [2], adding morbidity and mortality [3].

Reducing tidal volumes (*V*_T_s) below 6 mL/kg of ideal body weight could potentially decrease the cyclic stretch imposed on the lung [4, 5]. Conversely, excessively low *V*_T_s have the potential to lead to clinically significant hypercapnia-related acidosis [6] with harmful side effects [7, 8].

In this scenario, high-frequency oscillatory ventilation (HFOV) has been tested, because of its ability to provide adequate gas exchange even at very low tidal volumes [9–13]. This technique, however, may be cumbersome because it requires a dedicated ventilator and special training. Additionally, the *V*_T_ delivered can be susceptible to variations in airway resistance such as that which occurs with lung secretions [14]. Last but not least, it requires the use of high airway pressures, which may have deleterious effects, especially on the right ventricle [15]. Recently, two clinical studies in ARDS patients showed neutral [16] or disappointing [17] results in terms of mortality when HFOV was compared to a conventional mechanical ventilation strategy.

An alternative approach could be to apply moderately high frequency positive-pressure ventilation (HFPPV) using conventional mechanical ventilators. A similar strategy was explored in the 1980s [18, 19], but with special ventilators and before the well-established recognition of the importance of lung-protective strategies. HFPPV consists of applying respiratory rates intermediate between those used conventionally (≤ 35 breaths/min) and those used during HFOV (180 to 800 breaths/min). Potential advantages over HFOV would be the possibility to control the *V*_T_ delivered, the use of conventional mechanical ventilators obviating the need for specialized training, and maintenance of a low mean airway pressure. In this feasibility study, we tested in a swine model of ARDS whether such a strategy could result in *V*_T_ below 6 mL/kg while avoiding further increases in the partial pressure of arterial carbon dioxide (PaCO_2_) and maintaining a reasonable mean airway pressure (*P*_mean_).

## Methods

This study was approved by the Institutional Animal Research Ethics Committees of Hospital Sírio Libanês and of Faculdade de Medicina da Universidade de São Paulo, both in São Paulo, Brazil, and was performed according to the National Institutes of Health (USA) guidelines for the use of experimental animals. The experiments were done in eight previously healthy Agroceres pigs.

### Instrumentation

The animals were fasted overnight before the experiment with free access to water. They received an intramuscular injection of midazolam (0.3 mg/kg; Dormonid®, Roche, São Paulo, Brazil) and acepromazine (0.5 mg/kg; Acepran®, Andrômaco, São Paulo, Brazil). Through an auricular vein, anesthesia was induced with thionembutal (12 mg/kg; Tiopental®, Abbott, São Paulo, Brazil) and muscular relaxation with pancuronium bromide (0.1 mg/kg; Pavulon®, AKZO Nobel, São Paulo, Brazil). They were then submitted to tracheal intubation (cuffed 7.5-French cannula) and connected to the Servo-300 mechanical ventilator (Maquet, Rastatt, Germany) with the following parameters in a volume-controlled mode: tidal volume of 8 to 10 mL/kg, positive end-expiratory pressure (PEEP) of 5 cmH_2_O, inspiratory fraction of oxygen (FiO_2_) adjusted to keep arterial saturation between 94% and 96%, and respiratory rate (RR) necessary to keep PaCO_2_ between 35 and 45 mmHg. Anesthesia was maintained during the study period with midazolam (0.3 mg/kg/h) and fentanyl citrate (5 μg/kg/h; Fentanyl®, Janssen-Cilag, São Paulo, Brazil) and muscular relaxation with pancuronium bromide (0.2 mg/kg/h). The adequate depth of anesthesia during the surgical period was evaluated with maintenance of physiological variables (heart rate and arterial pressure) and absence of reflexes (corneal and hind limb flexion response), as well as unresponsiveness to stimuli during manipulation. Supplementary boluses of 3 to 5 μg/kg of fentanyl and 0.1 to 0.5 mg/kg of midazolam were administered as necessary. A continuous drip of 1,000 mL/h of Lactated Ringer’s solution was infused until the end of the induction of pulmonary injury, and then a continuous infusion of 5 mL/kg/h of Lactated Ringer was maintained until the end of the study.

Monitoring with continuous electrocardiography, oxymetry, and blood pressures was done with a multiparametric monitor (Dixtal-Philips DX 2020, São Paulo, Brazil). The left femoral artery was cannulated for blood pressure monitoring and blood sampling. The right internal jugular vein was cannulated with a 9-French introducer sheath (Arrow, Reading, PA, USA) through which a pulmonary artery catheter (Edwards Lifesciences, Irvine, CA, USA) was introduced for monitoring of the mean pulmonary artery pressure (PAPm), cardiac output, central venous pressure (CVP), and mixed venous blood gases (SvO_2_). A central venous catheter was introduced in the left internal jugular vein. A surgical cystostomy was done to quantify the urine output. The animal was connected to the NICO_2_ device (Novametrix Medical Systems, Wallingford, CT, USA) for airway end-tidal pressure of carbon dioxide (EtCO_2_), tidal volume, airway pressures, and airway flow monitoring.

The regional ventilation was monitored with electrical impedance tomography (EIT; Dixtal-Philips, São Paulo, Brazil) [20, 21]. The lungs were split in sternal and vertebral regions of the same height. The amount of ventilation to the regions studied was reported according to the ventilator settings used. Arterial blood gas analyses were done with the ABL 800 device (Radiometer, Copenhagen, Denmark). After the surgical period, the animals were allowed to rest for 60 min prior to the baseline data acquisition.

### Measurements

In all the steps of the study, the following data were collected:

1. Hemodynamic: heart rate, cardiac output, CVP, mean systemic arterial blood pressure (ABPm), PAPm, pulmonary artery occlusion pressure (PAOP), SvO_2_, and norepinephrine use and dosage
2. Respiratory: arterial partial pressure of oxygen (PaO_2_), PaCO_2_, EtCO_2_, *V*_T_, airway peak pressure (*P*_peak_), airway plateau pressure (*P*_plateau_) through expiratory valve occlusion after 2 s of inspiratory pause, intrinsic positive end-expiratory pressure (PEEPi) through expiratory valve occlusion after 4 s of expiratory pause, extrinsic positive end-expiratory pressure (PEEPe), mean airway pressure (*P*_mean_), inspiratory flow, inspiratory time (*T*_insp_), and ventilatory distribution EIT data
3. Metabolic: pH, lactate, temperature, and fluid balance

### Calculated variables

To obtain the calculated variables, we used the following formulas:

- Cardiac index (CI) = Cardiac output/Weight
- Systemic vascular resistance index = (ABPm − CVP) × 80/CI
- Pulmonary vascular resistance index = (PAPm − PAOP) × 80/CI
- Blood oxygen content (C × O_2_) = P × O_2_ × 0.0031 + 1.36 × Hb × S × O_2_
- Minute ventilation = *V*_T_ × RR
- PEEPtotal = PEEPi + PEEPe
- Alveolar oxygen partial pressure (P_A_O_2_) = 643 × FiO_2_/100 − (PaCO_2_/0.8)
- Alveolar-arterial oxygen [(A-a)O_2_] gradient = P_A_O_2_ − PaO_2_
- Pulmonary capillary oxygen content (CcO_2_) = PAO_2_ × 0.0031 + 1.36 × Hb
- Pulmonary shunt = (CcO_2_ − CaO_2_) × 100/(CcO_2_ − CvO_2_)
- Static compliance (*C*_static_) = *V*_T_/(*P*_plateau_ − PEEPtotal)
- Dynamic compliance (*C*_dyn_) = *V*_T_/(*P*_peak_ − PEEPtotal)
- Resistance (*P*_peak_ − *P*_plateau_)/Inspiratory flow

### ARDS induction

After the baseline data collection, ARDS was induced with repeated whole-lung lavage using 1 L of isotonic saline (37°C) until the PaO_2_ was below 100 mmHg for at least 10 min. Lung injurious ventilation was then started with the animal ventilated in pressure control mode with PEEP = 3 cmH_2_O, FiO_2_ = 1, inspiratory/expiratory time ratio (I/E) = 1:1, *P*_peak_ = 42 cmH_2_O, and a RR of 20 to 30 breaths/min [22]. Arterial blood gases were obtained every 15 min, and the PEEP could be increased up to 19 cmH_2_O targeting a PaO_2_ level between 55 and 80 mmHg, whereas the inspiratory pressure was limited at 48 cmH_2_O. The injurious ventilation was maintained until one of the following parameters was reached:

- An interval of 240 min of injurious ventilation
- A PAPm > 50 mmHg
- A *C*_static_ < 10 mL/cmH_2_O (with a PEEP = 10 cmH_2_O and *V*_T_ = 6 mL/kg)
- A PEEP persistently ≥ 15 cmH_2_O for at least two consecutive arterial blood sample analyses
- An ABPm < 70 mmHg in spite of the use of norepinephrine in a dosage higher than 0.5 μg/kg/min

After lung injury induction, the stabilization step started. The animal was ventilated according to the recommendations of the interventional group in the "ARMA" study [5] in a volume-controlled mode, with *V*_T_ = 6 mL/kg, RR = 35 breaths/min (the maximal respiratory rate allowed by the protocol - because of hypercapnia and acidosis), initial PEEP = 10 cmH_2_O (mean value used in the ARMA study), and FiO_2_ = 1.

An arterial blood sample was obtained every 10 min. Subsequently, PEEP and FiO_2_ were titrated according to the ARMA study PEEP table (aiming at a PaO_2_ = 55 to 80 mmHg) [5]. *V*_T_ and RR were kept constant during the stabilization step with no attempt to correct the PaCO_2_ level.

### Experimental protocol

After reaching a PaCO_2_ equilibrium (variation < 5% in three consecutive arterial blood samples), we considered that the stabilization phase was finished. The same PEEP level titrated at this time was used in the following steps of the study.

**Figure 1:**
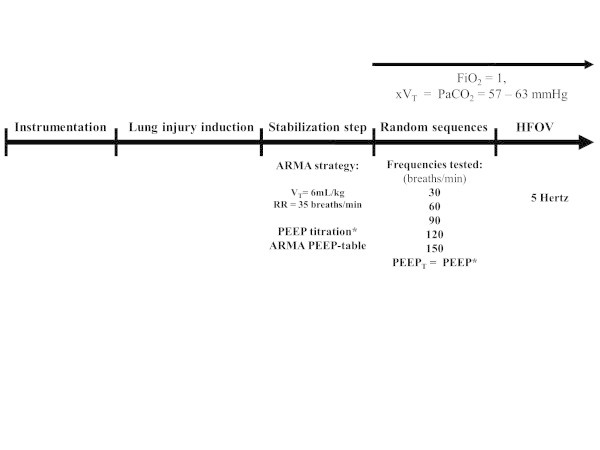
**Timeline of the study.** PEEP_T_, total end-expiratory positive pressure (intrinsic end-expiratory positive pressure plus extrinsic end-expiratory positive pressure should be the same as the end-expiratory positive pressure titrated following the ARMA PEEP table); *V*_T_, tidal volume; FiO_2_, inspiratory fraction of oxygen; PaCO_2_, partial arterial carbon dioxide pressure.

Four sequences of five different RRs were randomly tested, and these sequences were chosen to alternate higher and lower respiratory frequencies (Figure 1). The five RRs (ventilatory modes) randomized were as follows: RR = 30, 60, 90, 120, 150 breaths/min. At each sequence, *V*_T_ was adjusted to reach a PaCO_2_ target of 57 to 63 mmHg.

The randomization was done using sealed envelopes containing the proportion of 1:1:1:1 of the following RR sequences:

- Sequence 1 (60, 150, 90, 120, 30)
- Sequence 2 (90, 30, 120, 60, 150)
- Sequence 3 (120, 150, 90, 30, 60)
- Sequence 4 (150, 90, 30, 120, 60)

During this part of the protocol, the animals were ventilated using volume control ventilation, with FiO_2_ = 1 and square inspiratory flow = 1 L/s. At each step, PEEPi was measured every 10 min, and PEEPe was corrected in order to keep the PEEPtotal equal to the PEEP obtained during the equilibrium step using the ARMA PEEP table.

After completion of these randomized sequences, the animals were submitted to HFOV (Figure 1) at 5 Hz (SensorMedics 3100B, Yorba Linda, CA, USA) with a *P*_mean_ set at 30 cmH_2_O, I/E = 1:2, bias flow = 30 L/min, and the initial pressure amplitude = 80 cmH_2_O [9]. Unlike the other five RRs, of which the sequence was randomized, HFOV was always performed last, because of its higher *P*_mean_, which could induce lung recruitment.

An arterial blood sample was obtained each 10 min throughout the remainder of the study. After *V*_T_ changes or after pressure amplitude changes during HFOV, we waited until there were three consecutive measurements with the PaCO_2_ levels stable between 57 and 63 mmHg. Data were then collected, and the next step of the sequence was started. Between consecutive steps, a 40-s disconnection from the ventilator was done in order to avoid the carryover of the time-dependent alveolar recruitment. At the end of the experiments, the anesthesia was deepened with propofol overdose, and the animals were euthanized with a bolus of 10 mL of potassium chloride 19.1%.

### Statistical analysis

The Shapiro-Wilk goodness-of-fit model showed a non-parametrical distribution for most variables; therefore, data are reported as median [P25th,P75th]. Wilcoxon’s signed rank test was used to test variables before and after lung injury induction and to compare the upper and lower regional ventilation with the EIT. In order to avoid type I error, a modified Bonferroni’s correction was used to account for the multiple comparisons between upper and lower regions of ventilation. Therefore, the *p* value considered significant was 0.007 when comparing upper and lower regions during the various frequencies studied and 0.012 when comparing the effects of inspiratory pauses and the alveolar recruitment with a RR of 60 breaths/min. The analysis of variance for repeated measures on ranks (Friedman’s test) was used for analyses during the ventilatory modes tested. The *post hoc* analyses were done using Student-Newman-Keuls’ test. A *p* < 0.05 was considered significant. The analyses and graphs were done with the SigmaPlot 12.0 statistical package software (Systat Software, Inc. San Jose, CA, USA).

## Results

Eight pigs weighing 34 [29,39] kg were used. ARDS was induced using 10 [7,16] L of normal saline followed by injurious mechanical ventilation for 210 [40,225] min. The respiratory variables at baseline and after the induction of lung injury are shown in Table 1.

**Table 1:** **Respiratory variables at baseline and after the induction of lung injury**

Variable	Baseline	After lung injury
P**/**F ratio (mmHg)	427 [368,473]	97 [67,130]*
Shunt (%)	13 [12,15]	23 [16,32]*
Tidal volume (sternal)	4.8 [3.7,5.9]	4 [4.5,3.5]
Tidal volume (ventral)	4.2 [3.0,5.4]	2.5 [2,3]
*C*_static_ (mL/cmH_2_O)	27 [15,30]	12 [9,14]
Resistance (cmH_2_O/L/s)	8 [7,10]	18 [14,26]*

Values are presented as median [P25th,P75th] *C*_static_ and P/F denote static compliance and the ratio of arterial oxygen concentration to the fraction of inspired oxygen, respectively. *p < 0.05 vs baseline, Wilcoxon’s signed rank test.

**Figure 2:**
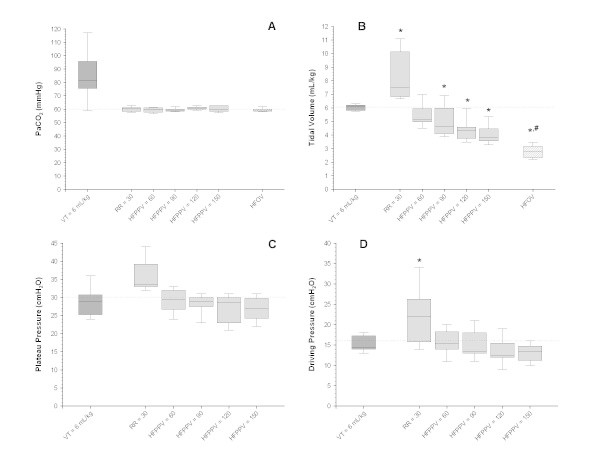
**Respiratory variables during the ventilatory modes tested. (A)** PaCO_2_ (mmHg; Friedman’s test, *p* = 0.011). **(B)** Tidal volume (mL/kg; Friedman’s test, *p* < 0.001). **(C)** Plateau pressure (cmH_2_O; Friedman’s test, *p* < 0.001). **(D)** Driving pressure (cmH_2_O; Friedman’s test, *p* < 0.001). *V*_T_, RR, HFPPV, and HFOV denote tidal volume, respiratory rate, high-frequency positive-pressure ventilation, and high-frequency oscillatory ventilation, respectively. The whiskers denote the P10th and P90th. *Student-Newman-Keuls’ *post hoc* analysis, *p* < 0.05 vs stabilization step (*V*_T_ = 6 mL/kg and RR = 35 breaths/min); ^#^Student-Newman-Keuls’ *post hoc* analysis, *p* < 0.05 vs HFPPV = 150.

**Figure 3:**
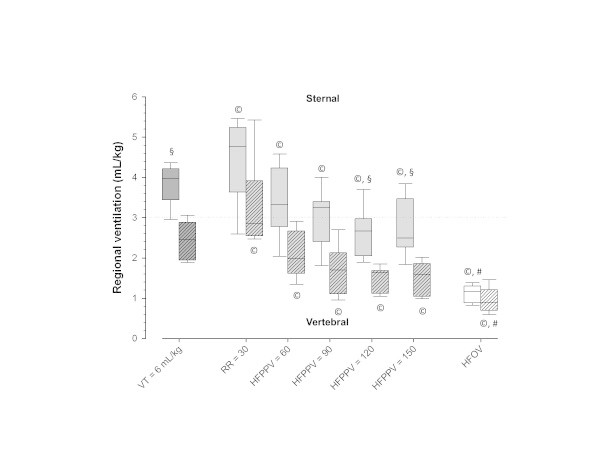
**Regional ventilation (mL/kg) in the sternal and vertebral portions of the thorax measured through EIT.** ^©^Student-Newman-Keuls’ *post hoc* analysis, *p* < 0.05 vs stabilization step (*V*_T_ = 6 mL/kg and RR = 35 breaths/min) (Friedman’s test, *p* < 0.001); ^#^Student-Newman-Keuls’ *post hoc* analysis, *p* < 0.05 vs HFPPV = 150 (Friedman’s test, *p* < 0.001); ^§^Wilcoxon’s test, *p* < 0.007 (Bonferroni’s correction for multiple comparisons) vs the vertebral region (gravitational-dependent region).

**Figure 4:**
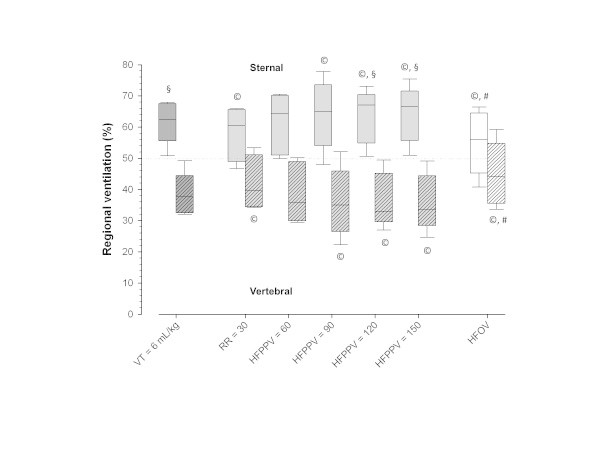
**Distribution of regional ventilation (%) in the sternal and vertebral portions of the thorax measured through EIT.** ^©^Student-Newman-Keuls’ *post hoc* analysis, *p* < 0.05 vs stabilization step (*V*_T_ = 6 mL/kg and RR = 35 breaths/min) (Friedman’s test, *p* < 0.001); ^#^Student-Newman-Keuls’ *post hoc* analysis, *p* < 0.05 vs HFPPV = 150 (Friedman’s test, *p* < 0.001); ^§^Wilcoxon’s test, *p* < 0.007 (Bonferroni’s correction for multiple comparisons) vs the vertebral region (gravitational-dependent region).

The FIO_2_ during the stabilization step was 0.7 [0.5,0.9]. The time to PaCO_2_ equilibrium was similar in the different phases of the experiment and equal to 50 [40,75] min. The most important respiratory data with different RRs are shown in Figure 2 and Table 2. During the stabilization step, PaCO_2_ was 81 [78,92] mmHg. In all other experimental phases, the PaCO_2_ was kept in the planned range of 57 to 63 mmHg (Figure 2A). *V*_T_ could be progressively reduced with increasing RRs (Figure 2B), as did regional ventilation (Figure 3). The ventilation to the dependent parts of the lung reduced to a greater extent leading to an increase in the sternal/vertebral ratio of regional ventilation (Figure 4). Additionally, low values of plateau and driving pressures were maintained at all RRs (Figure 2C,D, respectively). The HFOV led to the highest oxygenation, the lowest *V*_T_, and the most homogeneous distribution of ventilation (Table 2, Figures 2B and 4, respectively).

**Table 2:** **Respiratory variables through the ventilatory modes tested**

Variable	***V***_T_ = 6 mL/kg	RR = 30	HFPPV = 60	HFPPV = 90	HFPPV = 120	HFPPV = 150	HFOV	***p*** value ^a^
P/F ratio (mmHg)	95 [87,105]	151 [117,181]^b^	141 [102,189]^b^	132 [95,169]^b^	111 [86,162]^b^	112 [90,171]^b^	193 [146,216]^b,c^	P = 0.003
Gradient (A-a)O_2_	480 [465,493]	396 [383,452]^b^	427 [378,468]^b^	427 [394,466]	455 [406,481]	458 [394,478]	365 [350,420]^b,c^	P = 0.014
Minute ventilation (L/min)	6.9 [6.6,8.8]	8.7 [7.4,10.0]	11.1 [11.0,11.5]^b^	15.6 [14.0,17.5]^b^	18.6 [13.0,19.2]^b^	20.2 [19.5,21.3]^b^	27 [23.5,28.4]^b,c^	P < 0.001
Shunt (%)	43 [41,45]	29 [26,34]^b^	34 [30,40]	31 [28,44]	34 [31,45]	38 [30,43]	27 [25,32]^b,c^	P = 0.003
EtCO_2_ (mmHg)	58 [52,60]	43 [32,47]^b^	41 [37,49]^b^	40 [30,45]^b^	40 [31,48]^b^	34 [28,36]^b^	27 [23,30]^b,c^	P < 0.001
*C*_static_ (mL/cmH_2_O)	12 [10,14]	12 [9,14]	10 [9,13]	10 [9,12]^b^	10 [7,11]^b^	9 [8,11]^b^	-	P = 0.001
*C*_dyn_ (mL/cmH_2_O)	8 [7,9]	9 [6,10]	7 [6,9]	7 [6,8]	6 [5,7]^b^	6 [5,7]^b^	-	P < 0.001
Resistance (cmH_2_O/L/s)	8 [8,10]	10 [9,12]^b^	9 [8,9]^b^	9 [8,9]^b^	8 [8,11]^b^	9 [8,13]^b^	17 [13,20]^b,c^	P < 0.001
PEEP total (cmH_2_O)	14 [11,17]	14 [10,17]	14 [10,17]	13 [10,16]	13 [10,17]	13 [10,17]	-	P = 0.744
PEEP intrinsic (cmH_2_O)	0	0	0	0 [0,1]	0 [0,1]	2 [1,3]^b^	-	P < 0.001
PEEP extrinsic (cmH_2_O)	14 [11,16]	14 [11,16]	13 [10,17]	13 [10,16]	13 [10,16]	12 [9,14]	-	P < 0.001
Peak pressure (cmH_2_O)	45 [44,48]	54 [47,58]^b^	44 [42,47]	44 [41,45]	41 [38,44]	41 [38,43]	59 [51,79]^b,c^	P < 0.001
*P*_mean_ (cmH_2_O)	17 [15,20]	18 [16,22]^b^	18 [15,22]^b^	20 [18,23]^b^	20 [18,24]^b^	20 [17,23]^b^	29 [28,30]^b,c^	P < 0.001
Inspiratory flow (L/s)	1	1	1	1	1	1	-	P = 1.000
*T*_insp_/*T*_tot_ (%)	15 [14,17]	19 [14,22]	24 [20,28]^b^	34 [30,37]^b^	38 [33,44]^b^	42 [37,49]^b^	56 [50,67]^b,c^	P < 0.001

Values are presented as median [P25th,P75th]. ^a^The *p* value was obtained through Friedman’s test; ^b^Student-Newman-Keuls’ *post hoc* analysis, *p* < 0.05 vs *V*_T_ = stabilization step (*V*_T_ = 6 mL/kg and RR = 35 breaths/min); ^c^Student-Newman-Keuls’ *post hoc* analysis, *p* < 0.05 vs HFPPV = 150.

Only one animal needed norepinephrine during HFPPV, and the dose varied between 2.4 μg/kg/min (HFPPV = 60) and 3.2 μg/kg/min (HFOV). The hemodynamic and metabolic data with different RRs are shown in Table 3. Of note, the stabilization step with *V*_T_ = 6 mL/kg and RR = 35 breaths/min was associated with higher pulmonary artery pressures and lower pH.

**Table 3:** **Hemodynamic and metabolic variables during the ventilatory modes studied**

Variable	***V***_T_ = 6 mL/kg	RR = 30	HFPPV = 60	HFPPV = 90	HFPPV = 120	HFPPV = 150	HFOV	***p*** value ^a^
**Hemodynamic**								
Heart rate (bpm)	144 [125,165]	165 [124,182]	173 [144,181]	169 [130,189]	164 [128,196]	173 [142,196]	145 [122,155]	P = 0.210
Cardiac index (mL/kg/min)	138 [128,153]	126 [121,145]	145 [120,169]	127 [115,158]	141 [118,166]	132 [116,168]	126 [101,142]	P = 0.363
SV (mL)	28 [26,42]	27 [24,41]	27 [26,35]	26 [23,44]	27 [26,34]	30 [26,35]	31 [22,44]	P = 0.916
ABPm (mmHg)	90 [75,107]	86 [75,112]	84 [72,97]	91 [77,100]	83 [70,112]	78 [69,105]	82 [72,98]	P = 0.320
PAPm (mmHg)	43 [38,52]	34 [31,37]^b^	34 [28,36]^b^	36 [33,37]^b^	33 [30,47]^b^	38 [30,43]^b^	31 [30,40]^b^	P = 0.018
CVP (mmHg)	9 [9,12]	8 [7,12]	8 [6,10]	9 [6,10]	8 [7,10]	9 [7,10]	11 [10,12]^c^	P = 0.017
PAOP (mmHg)	12 [11,15]	12 [11,15]	12 [9,14]	12 [10,14]	12 [10,15]	12 [10,15]	14 [12,17]	P = 0.042
SvO_2_ (mmHg)	54 [47,70]	70 [49,79]	68 [48,71]	63 [48,66]	64 [43,74]	65 [55,73]	65 [50,75]	P = 0.140
SVRI (dynes.s^-1^(cm^5^)^-1^.kg^-1^	51.8 [41.8,56.4]	47.1 [39.8,65.6]	47.0 [35.0,50.9]	52.7 [36.7,61.4]	41.7 [33.4,63.6]	42.4 [28.8,66.5]	50.4 [31.6,55.8]	P = 0.558
PVRI (dynes.s^-1^.(cm^5^)^-1^.kg^-1^	22.3 [17.5,25.7]	15.7 [13.0,16.8]	15.6 [10.9,17.7]	16.7 [13.9,17.7]	14.2 [12.4,20.8]	16.8 [12.0,20.7]	13.6 [10.9,18.2]	P = 0.133
**Metabolic**								
Lactate (mEq/L)	1.7 [1.1,2.1]	1.3 [0.8,1.7]	1.6 [0.8,2.0]	1.7 [1.1,2.0]	1.4 [1.0,1.9]	1.6 [0.9,2.3]	1.5 [1.1,1.8]	P = 0.762
pH	7.13 [7.08,7.2]	7.25 [7.24,7.33]^b^	7.25 [7.24,7.35]^b^	7.26 [7.23,7.33]^b^	7.27 [7.2,7.3]^b^	7.26 [7.21,7.34]^b^	7.25 [7.2,7.32]^b^	P = 0.002
Temperature (°C)	38.6 [37.3,39.2]	39.7 [38.0,39.8]^b^	39.4 [37.6,39.6]^b^	39.2 [38.1,39.6]^b^	38.8 [37.6,39.6]^b^	39.0 [37.8,39.5]^b^	39.2 [38.1,39.8]^b^	P = 0.007
Fluid balance (mL)	−50 [−242,−5]	170 [101,278]	40 [−22,102]	100 [67,110]	60 [5,108]	40 [−58,88]	30 [−21,50]	P = 0.044

Values are presented as median [P25th,P75th]. ^a^The *p* value was obtained through Friedman’s test; ^b^Student-Newman-Keuls’ *post hoc* analysis, *p* < 0.05 vs *V*_T_ = stabilization step (*V*_T_ = 6 mL/kg and RR = 35 breaths/min); ^c^Student-Newman-Keuls’ *post hoc* analysis, *p* < 0.05 vs HFPPV = 150.

## Discussion

Our main finding was that, during protective mechanical ventilation of a severe ARDS swine model, the use of HFPPV with a conventional ventilator allows further reductions in *V*_T_ and PaCO_2_, leading to reductions in driving pressures and plateau pressures without increasing mean airway pressure. We did not identify any significant detrimental effect of the high RRs applied, even after careful assessment of hemodynamics, respiratory system mechanics, and gas exchange.

The possibility of further reducing the ventilator-associated lung injury is of utmost importance, with possible implications in terms of reducing death and multiple organ failure in ARDS patients [23]. Ventilation with low *V*_T_s (6 mL/kg) is still the standard support for those patients [5], although lower *V*_T_s might produce additional protection [6, 24]. Of note, one third of ARDS patients under protective ventilation still have lung hyperdistention, which is associated with increases in systemic inflammatory markers [25]. This subset of patients, usually more severely injured, could possibly benefit from further *V*_T_ reductions [24].

Increasing the RR at constant alveolar ventilation, we obtained a progressive decrease in *V*_T_s reaching levels below 4 mL/kg. This finding challenges the paradigm - promulgated by the design of many clinical trials that RRs should be kept equal to or less than 35 breaths per minute [5, 26–28]. In our model of severe ARDS, the standard of care [5] settings of *V*_T_s at 6 mL/kg and a maximum RR of 35 breaths/min led to a median PaCO_2_ value of 81 mmHg with a median pH of 7.13. Targeting a PaCO_2_ of 60 mmHg, we were able to reduce *V*_T_s by 36% with a RR of 150 breaths per minute. Other authors have shown, in an experimental model of ARDS, that higher RRs allow for a reduction in *V*_T_ when associated with a strategy to lower the dead space (aspiration of dead space) [29, 30]. Similarly, a recent study in patients with ARDS showed that protective *V*_T_ around 4 mL/kg can be achieved with modest increments in RR, provided that care is taken to minimize the circuit dead space [31]. These studies combined increases in RR with other measures to decrease the dead space. Our findings on the isolated effect of RR on the reduction of tidal volume help understand the independent effect of manipulating the RR.

The increases in RR were not associated with significant changes in gas exchange. We did notice a not significant but progressive fall in the median PaO_2_/FiO_2_ (P/F) ratio with increases in RR above 30 breaths per minute amounting to a fall of 26% at a RR of 150 breaths per minute (Table 2). Concurrently, the *T*_insp_/*T*_tot_ ratio increased from 19% to 42% when RR increased from 30 to 150 (Table 2), due to the fixed inspiratory flow rate and the need for higher minute ventilation at high RR. These increases in the *T*_insp_/*T*_tot_ ratio would favor a change in the P/F ratio in the opposite direction of the trend we found. These observations emphasize that with our relatively small sample size, we might have been underpowered to detect some differences such as the P/F ratio variation. If such trend proved significant in a larger study, it is possible that the lower tidal volumes at higher RR have favored the development of absorption atelectasis, although we cannot exclude that hemodynamic factors may played a role.

HFOV, a more classical strategy than HFPPV to provide adequate gas exchange at very low *V*_T_s [32, 33], has been recently shown to provide no benefit or even cause harm to patients with ARDS [16, 17]. Our results showed that HFOV = 5 Hz could stabilize the PaCO_2_ with *V*_T_s 26% lower than HFPPV = 150, however, with a RR twice as high and a *P*_mean_ 30% higher [15]. This is illustrative of the disproportionate increases in RR to maintain alveolar ventilation at progressively lower *V*_T_s, especially when close to the dead space, and the need to increase *P*_mean_, which may have deleterious hemodynamic effects. The consequence of this ventilation inefficiency might be an increased dissipation of energy in the lungs, potentially leading to more lung injury even at reduced stress and strain per breath. Therefore, reducing *V*_T_ without increasing mean airway pressure might be of special interest. In that sense, HFPPV might offer a better compromise between *V*_T_ and RR than HFOV.

Ventilation decreased more in the gravitation-dependent regions, a finding suggestive of reabsorption atelectasis, air trapping, or incomplete filling of those regions due to airway narrowing. Even after taking this ‘functional baby lung’ into account, the net result was likely a lesser degree of tidal lung stretch as suggested by the decrease in driving pressures and plateau pressures. Additionally, despite a preferential reduction in dependent ventilation (Figure 4), HFPPV could result in lower regional *V*_T_ in non-dependent regions (Figure 3).

### Limitations

Our study has several limitations. First, the arbitrary choice of the target CO_2_ level during HFPPV can be criticized. The CO_2_ value can be a confounding factor of the ventilatory settings during ARDS ventilation, with some studies showing a protective [34, 35] and others a potentially deleterious role [36, 37]. We chose a narrow range of 57 to 63 mmHg to avoid such potential confounding effect and to avoid significant acidosis (pH < 7.15), a goal we achieved in all experimental conditions. Likely, the main findings of the study would maintain had a normocapnia target been applied. Second, our study design, with sequential changes in the ventilator settings, was susceptible to carryover phenomena. We tried to avoid that effect through the randomization of sequences, the disconnection from the ventilator between the steps, and through a prolonged wait to the PaCO_2_ equilibrium. Third, the performance of conventional ventilators declines at very high RRs and low *V*_T_s, especially if low-compliance tubing is not employed [38]. Fourth, we did not rule out that histological damage to the lungs might have happened at those very high RRs. Fifth, HFOV was the last step of the study due to logistic issues and at this time the animals had significant positive fluid balances. This could be one explanation why HFOV was not associated with hemodynamic alterations, even with the use of higher *P*_mean_. Finally, we cannot directly extrapolate these experimental findings to patients, who have longer time constants than pigs and might not tolerate RRs as high. Interestingly, those with the most severe lung injury tolerate better very high RR, because of their low time constants. Even so, in our experience, it is difficult to apply RR > 60 breaths per minute to patients without leading to intrinsic PEEP.

## Conclusions

In an animal model of severe ARDS, as compared to the standard protective ventilation, high-frequency positive-pressure ventilation delivered by a conventional ventilator allowed further reductions in tidal volume and in inspiratory pressures. As such, HFPPV could be a well-suited alternative in the treatment of severe ARDS with very low lung compliance, although its impact on lung inflammation still awaits evaluation.

## Abbreviations

ABPm: mean systemic arterial blood pressure
ARDS: acute respiratory distress syndrome
CcO2: pulmonary capillary oxygen content
Cdyn: dynamic compliance
CI: cardiac index
Cstatic: static compliance
CVP: central venous pressure
CxO2: blood oxygen content
EIT: electrical impedance tomography
EtCO2: airway end-tidal pressure of carbon dioxide
FiO2: inspiratory fraction of oxygen
HFOV: high-frequency oscillatory ventilation
HFPPV: high-frequency positive-pressure ventilation
I/E: inspiratory/expiratory time ratio
PaCO2: partial arterial carbon dioxide pressure
PaO2: partial pressure of arterial oxygen
PAOP: pulmonary artery occlusion pressure
PAPm: mean pulmonary artery pressure
PEEP: positive end-expiratory pressure
PEEPe: extrinsic positive end-expiratory pressure
PEEPi: intrinsic positive end-expiratory pressure
Pmean: mean airway pressure
Ppeak: airway peak pressure
Pplateau: airway plateau pressure
RR: respiratory rate
SvO2: mixed venous blood gases
Tinsp: inspiratory time
VT: tidal volumes.
